# An Isobolographic Analysis of the Antinociceptive Effect of *Salvia hispanica* L. in Combination with *Citrus × latifolia* in Rats

**DOI:** 10.3390/nu17111884

**Published:** 2025-05-30

**Authors:** Lilian Dolores Chel-Guerrero, Rolffy Ortiz-Andrade, Enrique Sauri-Duch, Emilio Piña-Betancourt, Luis Hebert-Doctor, Myrna Déciga-Campos

**Affiliations:** 1 Laboratorio de Farmacología, Facultad de Química, Universidad Autónoma de Yucatán, Mérida 97000, Mexico; ldch.guerrero7@gmail.com (L.D.C.-G.); luis.herbert@correo.uady.mx (L.H.-D.); 2 Laboratorio de Análisis Instrumental, Tecnológico Nacional de México, Instituto Tecnológico de Mérida, Mérida 97118, Mexico; enrique.sd@merida.tecnm.mx (E.S.-D.); le20081634@merida.tecnm.mx (E.P.-B.); 3 Sección de Estudios de Posgrado e Investigación, Escuela Superior de Medicina, Instituto Politécnico Nacional, Ciudad de México 07340, Mexico

**Keywords:** antinociception, synergism, *Salvia hispanica*, *Citrus × latifolia*, traditional medicine, inflammatory pain, antioxidant activity, medicinal plants, herb–drug interaction

## Abstract

This study aimed to evaluate the antinociceptive effect of *Salvia hispanica* L. seeds, *Citrus* × *latifolia* (Lime) juice, and the interaction of their combination in rats using the writhing test. Dose–response curves were constructed for an *n*-hexane extract of *S. hispanica* seeds (100–300 mg/kg; *p.o.*) and *C.* × *latifolia* juice (10–300 mg/kg; *p.o.*) administered individually or in combination to rats subjected to 1% acetic acid-induced writhing. Isobolographic analysis was used to assess the interaction between the combinations. Results showed that both medicinal plants exhibited dose-dependent antinociceptive effects. The antinociceptive effect of *C. × latifolia* (ED_50_ = 43.95 ± 1.9 mg/kg) exhibited greater potency than *S. hispanica* (ED_50_ = 112.9 ± 2.0 mg/kg). Their combination (1:1 ratio) showed a synergistic antinociceptive effect (Z_exp_ = 4.9 ± 0.6 mg/kg vs. Z_add_ = 83.5 ± 1.7 mg/kg). Both extracts were non-toxic, according to the OECD-423 test. Antioxidant activity may have contributed to the observed antinociceptive synergy. This study demonstrates that the synergistic antinociceptive effects suggest that combining *S. hispanica* and *C. × latifolia* may be a promising therapeutic approach for managing inflammatory and visceral pain with potential clinical utility.

## 1. Introduction

Recently, the World Health Organization (WHO) has been promoting the integration of traditional medicine into health systems as a strategy for people-centered care, emphasizing that its use must be grounded in scientific research to ensure effectiveness, potency, and safety [1]. However, while herbal remedies offer therapeutic potential, they also present safety concerns, including adverse reactions and interactions with drugs, herbs, or food. Such interactions can have both beneficial and harmful effects. Herbs with similar mechanisms of action may produce additive effects, increasing the risk of adverse outcomes, whereas herbs with complementary mechanisms of action may exhibit synergistic effects, thereby enhancing therapeutic efficacy [2].

For example, the combination of *Zingiber officinale* Rosc. (ginger) with lemon, orange, or grapefruit increased the absorption of gingerol, an anti-inflammatory and antioxidant phytocompound found in ginger [3]. Conversely, the combination of *Lavandula angustifolia* Moench (English lavender) with *Matricaria chamomilla* L. (chamomile), *Valeriana officinalis* L. (garden heliotrope), and *Melissa officinalis* L. (Lemon balm) can potentiate sedative effects, increasing the risk of central nervous system (CNS) depression [4].

These interactions stem from various mechanisms, including alterations in drug metabolism, pharmacokinetics, and pharmacodynamics. Unfortunately, comprehensive information regarding herb-related adverse reactions remains limited. Addressing this gap requires a thorough investigation of herb interactions, the underlying mechanisms, and strategies to guarantee the safe use of herbal remedies [2].

*Salvia hispanica* (chia) belongs to the Lamiaceae family and is an herbaceous plant native to Mexico, Guatemala, and Nicaragua. Chia seeds have a long history of consumption in Mayan and Aztec cultures, where they were incorporated into both beverages and culinary dishes. However, they have not been historically associated with specific medicinal properties. In central and southern Mexico, chia seeds are commonly used to prepare fresh beverages and are often mixed with cucumbers or lemons [5].

*Citrus × latifolia* (Persian Lime) is a member of the Rutaceae family and is a small hybrid tree that can grow up to 6 m in height. Its origin is unclear, but it is thought to be in Malaysia or the East Indian archipelago. Historically, Persian lime has been used for its antiseptic, carminative, diuretic, and digestive effects [6]. Both species are in high demand globally, with their consumption steadily increasing [7,8].

Our team previously identified the main natural compounds in the *n*-hexane, dichloromethane, and methanol extracts of *Salvia hispanica* L. used in this study. Alcohols and saturated and unsaturated fatty acids were identified in the *n*-hexane and dichloromethane extracts, and sugars, glycosides, and polyalcohols were identified in the methanol extract ([9], Supplementary Material); these differences could affect the anti-inflammatory activity of the extracts.

Also, the seeds of *S. hispanica* contain key metabolites with reported anti-inflammatory and antinociceptive properties, including oleic, linoleic, α-linolenic, γ-linolenic, and hexanoic fatty acids; polyalcohol mannitol; and phenolic compounds such as ferulic acid, caffeic acid, chlorogenic acid, gallic acid, ellagic acid, *p*-coumaric acid, cinnamic acid, rutin, apigenin, epicatechin, kaempferol, and quercetin. Similarly, *C. × latifolia* juice contains metabolites linked to anti-inflammatory and analgesic activities, including citric and malic acids and citrus flavonoids such as eriocitrin, hesperidin, narirutin, and neoeriocitrin [10,11,12]. Although the use of *Salvia* species for pain relief in traditional medicine is well documented, particularly in Mayan culture, this knowledge has been transmitted orally across generations and remains largely undocumented. In addition, chia’s chemical composition and technological properties give the plant a high nutritional potential. Chia is a good source of polyunsaturated fatty acids, namely omega-3 and omega-6, and soluble dietary fiber. It also contains an appreciable number of proteins and phytochemicals. The nutritional value of chia is the reason why it is used in the prophylaxis of several diseases such as obesity, hypertension, cardiovascular diseases, cancer, and diabetes [9,10,13,14].

To date, to the extent of our literature review, the antinociceptive properties of *S. hispanica* and *C. × latifolia* juice, both as individual samples and in combination, remain scientifically unreported. This study aimed to evaluate the antinociceptive effect of *Salvia hispanica* (chia) seeds and *Citrus × latifolia* (Lime) juice, as well as the interaction of their combination, in rats using the writhing test, establishing the antioxidant activity of these species and the safety profile of the studied extracts.

## 2. Materials and Methods

### 2.1. Reagents

Diclofenac (75 mg) was used as a positive control since it belongs to the family of non-steroidal anti-inflammatory drugs (NSAIDs) and has high efficacy in inflammatory pain models; it was purchased from Novartis, Ciudad de México, Mexico). Acetic acid (ACS grade), *n*-hexane (HPLC grade), potassium chloride (KCl, analytical grade), magnesium sulfate (MgSO_4_; analytical grade), and calcium chloride (CaCl_2_; analytical grade) were purchased from J.T. Baker (Estado de Mexico, Mexico). Sodium chloride (NaCl, ACS grade) was purchased from Merck (Mexico City, Mexico), potassium phosphate monobasic (KH_2_PO_4_, analytical grade) and anhydrous dextrose (C_6_H_12_O_6_; analytical grade) from Reproquifin (Estado de Mexico, Mexico), sodium bicarbonate (NaHCO_3_; reactive grade) from Analytica (Monterrey, Mexico), and ethylenediaminetetraacetic acid (C_10_H_16_N_2_O_8_; EDTA; analytical reactive) from Fermont (Monterrey, Mexico). All treatments (extracts or diclofenac) were freshly prepared in saline solution 0.9% (vehicle) and administered by oral gavage (*p.o*). An algesic compound, 1% acetic acid, was diluted in water and injected intraperitoneally (0.1 mL/100 g, i.p.). The Krebs Henseleit Solution composition was as follows (10 mM): NaCl, 69.5 g; KCl, 3.5 g; MgSO_4_, 2.9 g; KH_2_PO_4_, 1.6 g; CaCl_2_, 2.2; C_6_H_12_O_6_ 1.09 g; NaHCO_3_, 2.1 g; and EDTA, 0.01 g.

### 2.2. Plant Material and Extraction

Seeds of *Salvia hispanica* L. (ID: SF2G/90/2022) were purchased from a certified supplier (SuperFoods2Go) in Jalisco, Mexico, in June 2022. Upon arrival, the seeds were air-dried at an ambient temperature (25 °C) for three days. Subsequently, the dried seeds were ground into a fine powder using a blender (Oster, Monona, Dane, KS, USA) and then sieved to a particle size of 500 μm (Sieve #35, Fisher Scientific, Boston, MA, USA). Then, 400 g of powdered seeds underwent ascending maceration processes (three cycles, 24 h each) using 100% *n*-hexane, dichloromethane, and methanol (100 g/L). The resulting extracts were filtered using Whatman grade 1 filter paper and concentrated under reduced pressure (50 °C, 22 inHg) using a rotary evaporator (BUCHI R-300 Meierseggstrasse Flawil, Switzerland) coupled with a vacuum pump (Lab Companion VE-11, Billerica, MA, USA). To ensure the complete removal of residual solvents and water, the extracts underwent lyophilization at 0.02 mBar and −50 °C using a FreeZone 6 L Console Freeze Dryer (Labconco Corp., Kansas City, MO, USA). This process yielded the following extracts: *n*-hexane (67 g), dichloromethane (10 g), and methanol (6 g) [14].

Freeze-dried lemon juice was prepared at the Instituto Tecnológico de Mérida. Thirty *Citrus × latifolia* Tanaka fruits were harvested at their commercial ripening stage in May 2024 from a local orchard in Calkiní, Campeche, Mexico. Upon collection, the fruits were washed, cut using a stainless-steel knife, and manually squeezed to yield 463 g of fresh juice. This juice was then filtered through a commercial plastic strainer and subsequently lyophilized at 0.04 mBar and −45 °C for 48 h using a Labconco Model 6 freeze-dryer (Labconco Corp., Kansas City, MO, USA). The resulting lyophilized juice was pulverized and stored at −20 °C in airtight plastic bags until further analysis. All methodologies employed in this study adhered to previously standardized protocols [15].

Plant material authentication was conducted by Dr. Salvador Flores Guido of the Botany Department, Facultad de Veterinaria y Zootecnia, Universidad Autónoma de Yucatán (UADY). Voucher specimens were deposited at the UADY herbarium “Alfredo Barrera Marín” with Voucher No. J.S. Flores 12877.

### 2.3. Animals

Female Wistar rats (150–250 g body weight, inclusion criteria a priori) were used to evaluate antinociceptive activity, whereas male and female Wistar rats (180–250 g body weight, inclusion criteria *a priori*) were used for acute toxicity studies. We used 27 groups of female rats, with *n* = 6 for each group, for antinociceptive activity and 2 groups of female rats and 4 groups of male rats, with *n* = 3 for each group, for toxicity, for a total of 180 animals (168 female rats and 12 male rats). Based on the committee’s recommendations, we decided on the sample size and the protocol’s test recommendations. All animals were obtained from the Universidad Anáhuac Mayab Animal Facility, Mérida, Yucatán, Mexico. The rats were housed in polycarbonate cages under standard laboratory conditions, including a 12 h light/dark cycle, controlled temperature (25 ± 2 °C), and relative humidity (45–65%). They were fed a standard rodent diet and provided with water *ad libitum*. Each animal was marked with a numeric signal and color for differentiation and was selected for each test by simple randomization. Likewise, each animal box was labeled with a descriptive sheet that included information for traceability and to avoid potential confusion. This sheet included data such as origin, date of arrival, number of animals, sex, owner, and experiment. Each animal’s logbook was also kept (with a marker on the animal’s tail to prevent damage), which would be used for date, weight, type of experiment, treatment, and results obtained. Observations were recorded for each animal if any relevant details were found. The principal investigator was always aware of the group assignment at the different stages.

All experiments were conducted strictly following the ethical guidelines established by the Mexican Official Norm for Animal Care and Handling (NOM-062-ZOO-1999). Furthermore, animal studies were reported in compliance with the ARRIVE guidelines (UK), the Animals (Scientific Procedures) Act 1986 and associated guidelines, EU Directive 2010/63/EU on the protection of animals used for scientific purposes, and the National Research Council’s Guide for the Care and Use of Laboratory Animals. The experimental procedures adhered to the Guidelines on Ethical Standards for Investigation of Experimental Pain in Animals [16].

The study protocol was approved by the SIP-IPN (20253951-MDC) and the Institutional Animal Care and Use Committee of the Universidad Juárez Autónoma de Tabasco (protocol code UJAT-0001-2017). Based on the committee’s recommendations, we decided on the sample size and the protocol’s test recommendations. Efforts were made to minimize animal suffering and to reduce the number of animals used in the study. Animals were sacrificed by cervical dislocation after the experiments.

### 2.4. Study Design

The study began with an assessment of the safety of *S. hispanica* seeds and *C. × latifolia* fruit juice by determining the acute oral toxicity of *n*-hexane, dichloromethane, and methanol extracts of *S. hispanica*, as well as the freeze-dried juice of *C. × latifolia*, in accordance with the OECD test guidelines (180 animals were used; two groups of female rats and four groups of male rats, *n* = 3 for each group).

Subsequently, the antinociceptive activity of *S. hispanica* extracts (*n*-hexane, dichloromethane, and methanol) was assessed using an acetic acid-induced nociception test in 27 groups of female Wistar rats (*n* = 6 each group). Logarithmic doses (100 and 300 mg/kg) of each extract were evaluated to identify the most active extract. Following this, the extracts of both *S. hispanica* (the most active) and *C. × latifolia* were individually analyzed, along with five combinations of the extracts, in the writhing test in rats.

Isobolographic analysis was conducted to determine the pharmacological interactions between *S. hispanica* and *C. × latifolia*. Finally, to explore the potential mechanism of action of the observed antinociceptive effects, the antioxidant capacities of both *S. hispanica* and *C. × latifolia* were evaluated, and their total phenolic content was determined.

### 2.5. Acute Oral Toxicity in Rats

The half-lethal dose (LD_50_) was estimated using the fixed-dose method described in the Guidelines for the Testing of Chemicals (OECD) (Test No. 423: Acute Oral Toxicity-Class Method; OECD, 2001) in Wistar rats [17,18,19]. This procedure is reproducible, uses very few animals, and can rank substances similarly to the other acute toxicity testing methods (Test Guidelines 420 and 425). This test was adopted in March 1996 as the second alternative to the conventional acute toxicity test, described in Test Guideline 401. Briefly, three fixed doses (50, 300, and 2000 mg/kg) of the *n*-hexane, dichloromethane, and methanol extracts of *S. hispanica* seeds and the lyophilized juice of *C. × latifolia* fruit were orally administered to rats (*n* = 3). The same protocol was applied to the female rats at the highest dose (2000 mg/kg). An additional group was administered the vehicle as a control. A total of 27 Wistar rats were used (21 male and 6 female).

The body weights of all rats in each group were recorded, and the animals were closely monitored for toxic symptoms and behavioral changes, including sedation, hyperactivity, writhing, pruritus, feeding and drinking habits, general morphological changes, and leg bites. Observations were conducted for the first 60 min post-administration and periodically for 24 h and continued daily for 14 days.

### 2.6. Antinociceptive Effect Assessment in Wistar Rats by the Writhing Test

The initial series of experiments aimed to identify the most effective *S. hispanica* seed extracts for use in combination with *C. × latifolia*. Eight groups of rats, each consisting of six animals, received various treatments by oral administration, including a vehicle (0.9% saline solution); *n*-hexane, dichloromethane, and methanol extracts of *S. hispanica* at doses of 100 and 300 mg/kg; and diclofenac sodium (100 mg/kg) as a positive control. One hour after the treatment, the rats were subjected to a nociceptive agent to induce pain-like behavior. This was achieved through an intraperitoneal injection of 1% acetic acid (0.1 mL/100 g), which irritates the peritoneal cavity and leads to characteristic pain-like responses. The observed behaviors included abdominal contractions and stretching of the hind limbs. The number of writhing movements (stretches) was recorded every five minutes over a total observation period of 60 min, following the administration of the nociceptive agent. The inhibition of the writhes for the positive control was taken as the standard and compared with the test of the samples and the control. A reduction in the number of writhes was interpreted as indicative of antinociceptive activity.

In the second set of experiments, 12 groups of rats (*n* = 6 per group) were used; the *n*-hexane extract of *S. hispanica*, which showed the highest activity level, was further evaluated at additional doses (10, 30, and 150 mg/kg, *p.o.*) to establish a complete dose–response curve. The freeze-dried juice of *C. × latifolia* fruit was also tested at 10, 30, 100, 150, and 300 mg/kg doses *p.o.* in the acetic acid-induced nociception model. Diclofenac (100 mg/kg) was used as a reference drug, and saline solution (0.9%) as a vehicle.

In the third series of experiments, seven groups of Wistar rats were used (*n* = 6 per group) for five combinations of extracts, which were administered according to the isobolographic method (Table 1). Diclofenac sodium (100 mg/kg) was used as a reference drug, and saline solution (0.9%) as a vehicle. These combinations were evaluated for their effects on acetic acid-induced nociception at different doses of *S. hispanica* and *C. × latifolia*.

### 2.7. Isobolographic Analysis of Interactions 

An isobologram was constructed to characterize the interaction between the hexane extract of *S. hispanica* seeds and freeze-dried *C. × latifolia* Tanaka fruit juice. The isobologram was constructed using values obtained as the effective dose 50 (ED_50_) from the extracts administered individually or in combination.

The theoretical additive doses (Z_add_) and their standard errors of the mean (SEMs) for each combination were calculated according to the method described by Tallarida using the following equation:

Z_add_ = fA + (1 − f)B

where A (*S. hispanica*) and B (*C. × latifolia*) are the ED_50_ values of the extracts in combination. For a 1:1 fixed ratio, f was 0.5, and (1 − f) was 0.5. Z_add_ is the total additive dose of the extracts, which theoretically provides a 50% reduction in the rats in the vehicle group. Here, Z_exp_ is the experimentally determined total dose of the mixture in combination. The Z_exp_ values (with their 95% confidence limits) were determined from the respective dose–response curves of combined drugs according to standard linear regression analysis of the log dose–response curve. Subsequently, the 95% confidence limits were transformed into SEMs. Statistical comparison of the experimentally determined (Z_exp_) with the theoretically calculated (Z_add_) values was performed using Student’s *t*-test, according to procedures previously described [20,21,22]. The isobologram was constructed by connecting the ED_50_ value of *S. hispanica* on the abscissa with the ED_50_ value of *C. × latifolia* on the ordinate to obtain an additive line. The amounts of each component in combination (experimental [Z_exp_] and theoretical additive [Z_add_] doses) were also plotted on the same graph. The theoretical additive point lies on a line connecting the ED_50_ values of individual extracts [20,21,22].

### 2.8. Quantification of Total Soluble Phenolic Compounds (TSPCs)

The total soluble phenolic compounds (TSPCs) were quantified using the Folin–Ciocalteu method as described by González-Aguilar et al. [23]. Absorbance was measured using a UV-VIS spectrophotometer (Cary 60, Agilent, Santa Carolina, CA, USA). The concentration of phenolic compounds was calculated from a calibration curve constructed using gallic acid (Sigma Aldrich, St. Louis, MO, USA) in the range of 50–1100 mg/L.

The results are expressed as milligrams of gallic acid equivalents (mg GAE) per gram of “dry” extract (freeze-dried extracts are known as “dry” extracts). Each measurement was performed in triplicate to ensure accuracy and reproducibility.

### 2.9. Antioxidant Capacity Analysis by Iron Reducing Power Using the Ferric Reducing Antioxidant Power Method

The antioxidant capacity through iron reducing power was determined using the Ferric Reducing Antioxidant Power method as described by Can-Cauich et al. [12]. The results were expressed as mg of Trolox equivalents (mg TE) per gram of “dry” extract, based on a calibration curve constructed with the absorbance of Trolox solutions at concentrations ranging from 20 to 320 mg/L. Each determination was performed in sextuplicate to ensure accuracy and reproducibility.

### 2.10. Antioxidant Capacity Analysis Using the 2,2-Diphenyl-1-Picrylhydrazyl (DPPH) Method

The antioxidant capacity was also assessed using the 2,2-diphenyl-1-picrylhydrazyl (DPPH) free radical method as described by Brand-Williams et al. [24]. Absorbance was measured at 515 nm after 30 min of reaction using an Agilent Technologies Cary 60 UV-Vis spectrophotometer (USA). The results were expressed as mg of Trolox equivalents (mg TE) per liter, calculated from a calibration curve developed with Trolox solutions at concentrations ranging from 20 to 320 mg/L. The determinations were performed in sextuplicate to ensure reliability.

### 2.11. Statistical Analysis

Dose–response curves were obtained from the time courses (time vs. number of writhes). The ED_50_ was determined using a logarithmic linear system (using the slope to establish the 50% effect). The standard error was determined from the standard deviation of the data, considering the sample size. Confidence limits were determined using a 95% confidence interval (Z = 1.96). All data were compared using one-way analysis of variance (ANOVA) followed by Dunnett’s or Tukey’s tests. For isobolographic analysis, Student’s *t*-test was applied. All analyses were performed using the GraphPad Prism software version 8.0.1 (San Francisco, CA, USA). *p* < 0.05.

## 3. Results

### 3.1. Acute Oral Toxicity

No signs of toxicity or mortality were observed following oral administration of *n*-hexane, dichloromethane, and methanol extracts of *S. hispanica* or freeze-dried juice of *C. × latifolia* fruit at 50, 300, and 2000 mg/kg. Behavioral assessments revealed no indications of pruritus, lethargy, excessive water consumption, or paw-biting. Additionally, no adverse effects such as anorexia, ataxia, hypoactivity, piloerection, or syncope were observed, body weight remained stable, and no gastric ulcers were observed during the examination. Consequently, the overall acute toxicity (LD_50_) of both samples, when administered orally to rats, was determined to be LD_50_ > 2000 mg/kg of body weight.

### 3.2. Antinociceptive Effect of n-Hexane, Dichloromethane, and Methanolic Extracts

**Figure 1:**
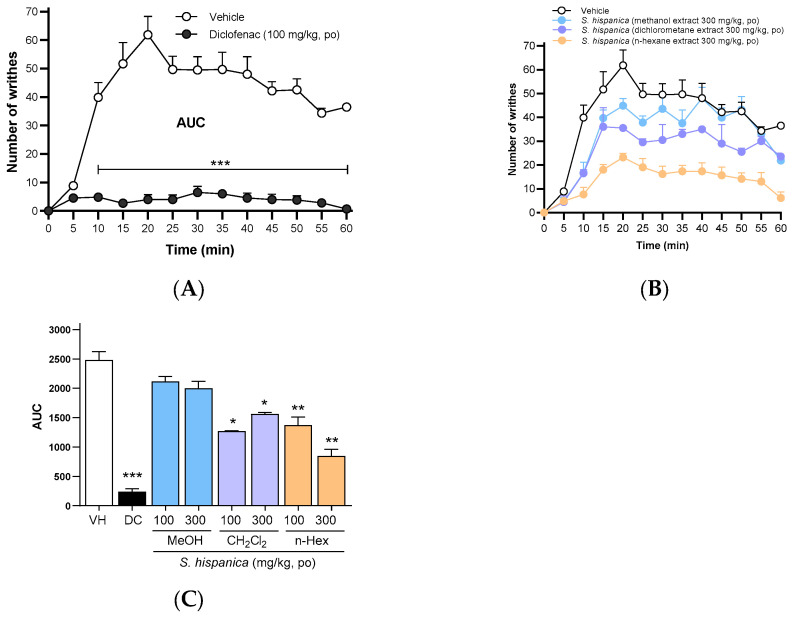
Experimental nociception test in female Wistar rats with 1% acetic acid intraperitoneal injection. Antinociceptive behavior was characterized by a decrease in the frequency of writhings (manifested as arching of the back, hind limb extension, and abdominal muscle contraction) over 60 min, measured every 5 min. (**A**) Temporal course of the number of writes vs. time of vehicle (saline solution 0.9%) and diclofenac (100 mg/kg *p.o.*) groups. (**B**) Temporal course of antinociceptive effect of *S. hispanica* seeds extracts (methanol [MeOH], dichloromethane [CH_2_Cl_2_], or *n*-hexane [*n*-Hex]) at 300 mg/kg administered orally 60 min before 1% acetic acid. (**C**) Bars represent the AUC of temporal courses of MeOH, CH_2_Cl_2_, or *n*-Hex at 100 and 300 mg/kg administered orally. Data are expressed as the mean ± SEM of 6 animals per group. * *p* < 0.05, ** *p* < 0.01, and *** *p* < 0.001 indicate statistical differences vs. the vehicle group, using a one-way ANOVA followed by the Dunnett test.

The antinociceptive effects of *n*-hexane, dichloromethane, and methanol extracts of *S. hispanica* seeds, evaluated using the writhing test in rats, are presented in Figure 1. Figure 1A depicts the temporal course of writhing behavior in rats after acetic acid and diclofenac sodium administration. A clear increase in the number of writhes is observed. However, when diclofenac (100 mg/kg) was administered, the writhing behavior was completely suppressed, confirming diclofenac’s potent antinociceptive effect. Figure 1B illustrates the antinociceptive effects of the three extracts at 300 mg/kg. Among the evaluated extracts, the *n*-hexane extract showed the highest antinociceptive activity in the writhing test. Figure 1C compares the effects of different doses of *n*-hexane, dichloromethane, and methanol extracts of *S. hispanica* seeds (100 and 300 mg/kg, *p.o*.). Notably, the *n*-hexane extract demonstrated the most significant antinociceptive effect, with a dose–response relationship. The dichloromethane extract exhibited a paradoxical decrease in antinociceptive activity with increasing doses. In contrast, the methanol extract showed no statistically significant difference between the tested doses (100 and 300 mg/kg, *p.o.*) and the vehicle, indicating the absence of antinociceptive activity.

### 3.3. Antinociceptive Dose–Response Curve of S. hispanica and C. × latifolia

**Figure 2:**
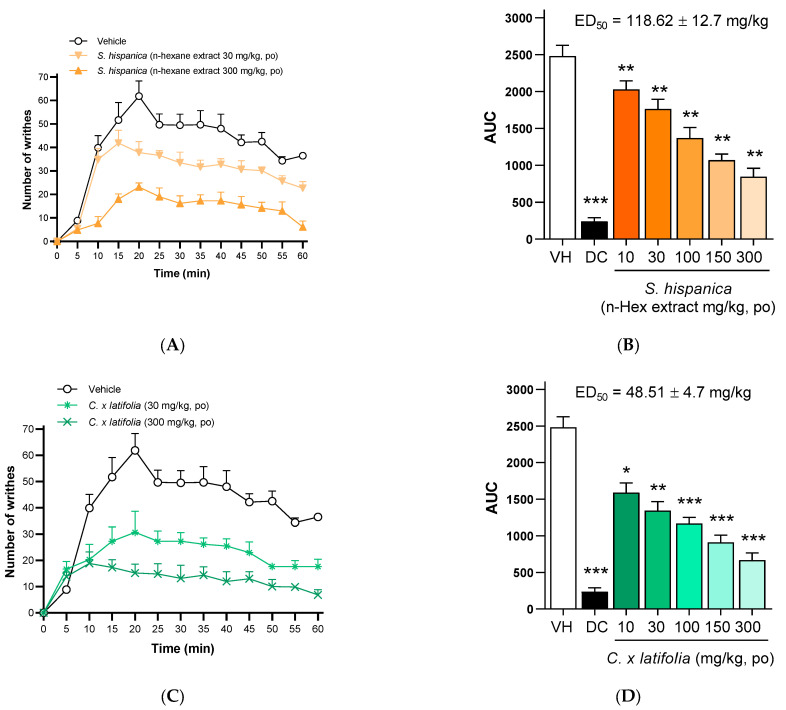
Temporal course of the antinociceptive effect of *n*-hexane extract of *S. hispanica* (30 and 300 mg/kg, *p.o.*) and *C. × latifolia* (30 and 300 mg/kg, *p.o.*) in nociception induced by acetic acid injection ((**A**) and (**C**), respectively). Dose–response curve expressed as AUC of *n*-hexane extract of *S. hispanica* (30 and 300 mg/kg, *p.o.*) and *C. × latifolia* (30 and 300 mg/kg, *p.o.*) in nociception induced by acetic acid injection ((**B**) and (**D**), respectively). Data are expressed as the mean ± SEM of 6 animals per group. * *p* < 0.05, ** *p* < 0.01, and *** *p* < 0.001 indicate statistical differences vs. the vehicle group, using a one-way ANOVA followed by the Dunnett test.

Figure 2A shows the temporal antinociceptive effects of the *n*-hexane extract of *S. hispanica* seeds at 30 and 300 mg/kg doses. The maximum effect was observed at 15 min for the 30 mg/kg dose and at 20 min for the 300 mg/kg dose. Subsequently, antinociceptive effects gradually decreased over time. Figure 2B shows the dose–response curve expressed as the area under the curve (AUC). From these data, the effective dose 50 (ED_50_ = 118.62 ± 12.7 [93.73–143.51] mg/kg, *p.o.*) of the *n*-hexane extract was calculated using a logarithmic–linear method. Similarly, the antinociceptive effect of freeze-dried *C. × latifolia* fruit juice was assessed. Figure 2C shows that the maximum effect occurred at 20 min for the 30 mg/kg dose and at 10 min for the 300 mg/kg dose, after which the effect gradually diminished. Using the dose–response data and a logarithmic–linear method (Figure 2D), the ED_50_ of the freeze-dried juice was determined to be 48.51 ± 4.7 [39.3–57.7] mg/kg, demonstrating that *C. × latifolia* juice was more potent than the *n*-hexane extract from *S. hispanica*. The calculated ED_50_ values were subsequently used to establish combinations for isobolographic analysis.

### 3.4. Isobolographic Analysis

**Figure 3:**
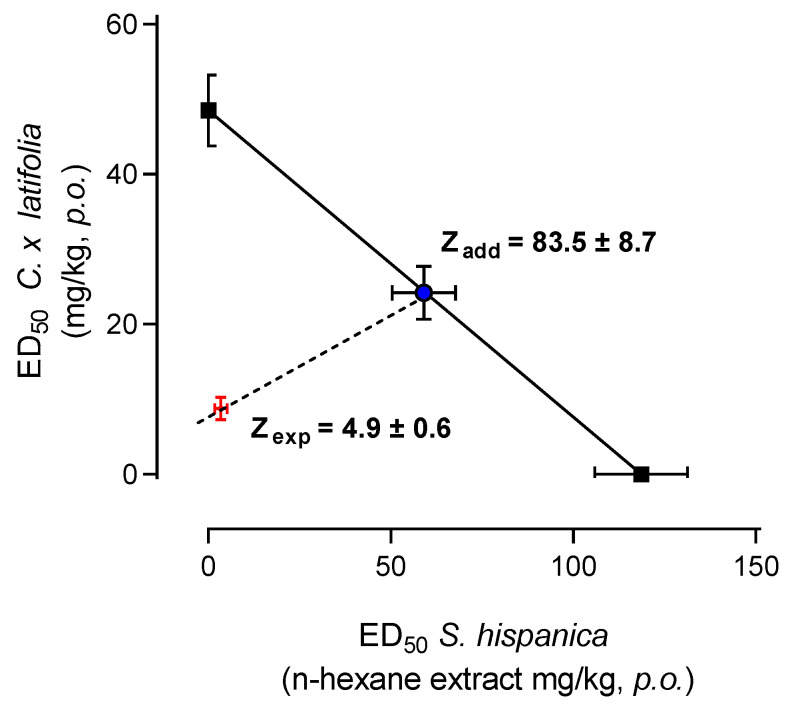
Isobologram showing the interaction of the fixed 1:1 ratio of the combination of *S. hispanica* + *C. × latifolia* in the writhing test in female Wistar rats. Horizontal and vertical bars indicate the SEM of at least six animals. The theoretical additive line is the oblique line between the x- and y-axis. The point in the middle of this line is the theoretical additive point calculated from the individual drug ED_50_ value. The experimental point is the observed ED_50_ value obtained with the above combination. The experimental ED_50_ point lies near the additive line, indicating an additive interaction.

The antinociceptive response to the coadministration of a fixed-ratio mixture (1:1) of the ED_50_ from the *n*-hexane extract of *S. hispanica* and the ED_50_ from the freeze-dried juice of *C. × latifolia* fruit was evaluated. Isobolographic analysis of this combination revealed an experimental ED_50_ = 4.9 ± 0.6 [3.72–6.07] mg/kg, which was significantly lower than that theoretically calculated as ED_50_ = 83.3 ± 8.7 [66.24–100.35] mg/kg (Figure 3).

**Figure 4:**
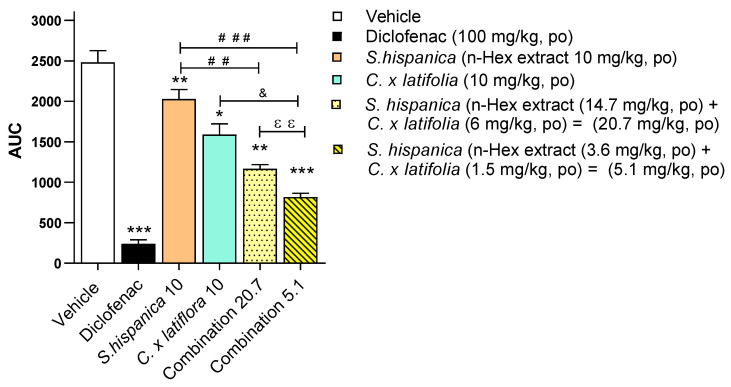
Antinociceptive effect in female Wistar rats with nociception-induced acetic acid expressed as AUC of *S. hispanica* (*n*-hexane extract 10 mg/kg, *p.o.*) + *C. × latifolia* (10 mg/kg, *p.o.*) administered individually compared with the combinations *S. hispanica* (*n*-hexane extract 14.7 mg/kg, p.o.) + *C. × latifolia* (6 mg/kg, *p.o.*) and *S. hispanica* (*n*-hexane extract 3.6 mg/kg, *p.o.*) + *C. × latifolia* (1.5 mg/kg, *p.o.*). Data are expressed as the mean ± SEM of 6 animals per group. * *p* < 0.05, Student′s *t*-test. ** *p* < 0.01 and *** *p* < 0.001 with respect to vehicle; ^##^ *p* < 0.01 and ^###^ *p* < 0.001 with respect to *S. hispanica*; ^&^ *p* < 0.05 with respect to *C. × latifolia*; ^ɛɛ^ *p* < 0.01 for both combinations. A one-way ANOVA was used, followed by the Tukey test.

Figure 4 illustrates an example of the potential antinociceptive effects of a combination of *S. hispanica* and *C. × latifolia*. At a dose of 10 mg/kg, the individual effects of both species showed modest antinociceptive responses (18.3% and 35.6%, respectively) when compared to the vehicle. However, when administered together at a combined dose of 20 mg/kg, the antinociceptive effect increased to 53%. Notably, when lower doses (5.1 mg/kg) of both plants were coadministered (comprising 3.6 mg of the *n*-hexane extract of *S. hispanica* and 1.5 mg of *C. × latifolia*), which individually exhibited minimal antinociceptive effects, the combination resulted in a substantial increase in antinociception, reaching 67%.

### 3.5. Antioxidant Capacity

*S. hispanica* and *C. × latifolia* exhibited total soluble phenolic compound concentrations of 6.6 ± 0.28 and 14.8 ± 1.97 mg equivalents to mg of gallic acid per gram of extract, respectively. The antioxidant activity of the samples differed significantly from that of the freeze-dried juice of *C. × latifolia* fruit, which demonstrated the highest antioxidant activity, as determined by the Ferric Reducing Antioxidant Power and 2,2-diphenyl-1-picrylhydrazyl methods (Table 2).

## 4. Discussion

Plant combinations have been used in several cultures, such as in traditional Chinese medicine, which has documented over 2000 years of plant–plant combinations. However, these combinations can result in complex effects owing to interactions among individual components. Information on plant–plant interactions remains scarce [25]. Since ancient times, *Salvia* and *Citrus* species have been used for their analgesic and anti-inflammatory properties. *Salvia hispanica* L. and *Citrus × latifolia* Tanaka are widely consumed worldwide and are often combined as beverages owing to their purported health benefits, including antioxidant and anti-diabetic properties. The preliminary study demonstrated that the *S. hispanica* extract exhibited a more pronounced antinociceptive effect than MeOH and CH_2_Cl_2_ extracts. It is important to note that multiple factors, including the experimental model, route of administration, and dose employed, influence the observed pharmacological activity. Therefore, additional studies are necessary to definitively assess the MeOH extract’s potential efficacy and rule out false-negative results due to these variables.

To explore this further, we assessed oral acute toxicity, antinociceptive effects, and the interaction between *S. hispanica* and *C. × latifolia* (It should be noted that no animal was excluded in these tests). Although the writhing test is not directly scalable to humans because rat and human physiology differ in how they perceive and respond to pain, it is a good screening tool for assessing analgesic agents. With this objective, we used it in the present study. In this writhing test in rats, *C. × latifolia* exhibited a dose-dependent antinociceptive effect with greater efficacy and potency than *S. hispanica*, as indicated by the ED_50_ values: *C. × latifolia* was 2.6 times more potent than *S. hispanica*. When coadministered in a fixed 1:1 ratio (based on ED_50_ values), *C. × latifolia* significantly enhanced the antinociceptive response of *S. hispanica* in a synergistic manner. This synergism is noteworthy because it reduces the dose while achieving higher efficacy, potentially minimizing adverse reactions. This observation also suggests that the mechanisms of action of *S. hispanica* may differ from those of *C. × latifolia*.

Pharmacological plant–plant interactions may modify biological processes that regulate mechanisms of action, absorption, metabolism, and elimination. Sometimes, the combination of plants does not generate synergistic interaction because their components can generate contrary mechanisms of action, or their effect can be limited to the occupation of receptors, resulting in adverse events [26,27]. Although the mechanisms underlying these interactions are not fully understood, in vitro studies suggest that enzymes from the cytochrome P450 (CYP) family, such as CYP1A2, CYP3A1/2, and CYP2E1, play a key role. These enzymes can mediate plant–plant interactions if one or both plants are metabolized primarily through CYP [25,26,27]. For instance, Wang et al. [28] reported that the combination of *Panax ginseng* and *Veratrum nigrum* induced the expression of CYP1A1. *P. ginseng* has an inhibitory effect on CYP2B1/2 and an inductive effect when used with *V. nigrum*. The combination of *P. gingseng* with *V. nigrum* also induced the expression of CYP3A1 [28].

However, beneficial therapeutic interactions may also occur, as evidenced by the potentiation of the antinociceptive effect observed with *S. hispanica* and *C. × latifolia*. Potentiation occurs when two plants with similar bioactivities are combined, with one acting as the principal and the other as an auxiliary plant, thus enhancing the effect of the principal. For example, Zhang et al. demonstrated a synergistic interaction between Paeonia root’s total glycosides and Glycyrrhiza’s total flavonoids using isobolographic analysis in a rat model of neuropathic pain [29].

Our team previously identified the main natural compounds in the *n*-hexane, dichloromethane, and methanol extracts of *S. hispanica* L. used in this study. Alcohols and saturated and unsaturated fatty acids were identified in the *n*-hexane and dichloromethane extracts, and sugars, glycosides, and polyalcohols were identified in the methanol extract. The first 20 natural products predicted from the results of the 13C NMR-based dereplication analysis in *n*-hexane *S. hispanica* extract were nonanoic acid, hexanol, capryc acid, undecanoic acid, heptanoic acid, amyl alcohol, hexanoic acid, undecane, octanol, 2-hexenol, 3-octenol, caprylic acid, myristoleic acid, linoleic acid, α-linolenic acid, arachidonic acid, methyl linolenate, methyl octadeca-9,12,15-trienoate, hexyl octanoate, and lauric acid. Of these products, only 3-octenol was not identified in the dichloromethane and methanol extracts. And in dichloromethane, heptanoic acid, hexanoic acid, and 2-hexenol were not identified either, but it had n-tridecanoic acid, myristic acid, oleic acid, and pentadecanoic acid. Among the natural products identified in *n*-hexane extracts, the methanol extracts only exhibited hexanol, undecanoic acid, heptanoic acid, and 2-hexenol [9]. These differences could affect the anti-inflammatory activity of the extracts.

Oleic acid, linoleic acid, α-linolenic acid, γ-linolenic acid, and hexanoic acid in the *n*-hexane extract ([9], Supplementary Material) and the primary components reported in Persian lemon fresh fruit or juice (*C. × latifolia*) as citric and malic acids and flavonoids such as eriocitrin, hesperidin, narirutin, and neoeriocitrin are known for their anti-inflammatory and analgesic properties, which likely contribute to the antinociceptive activity of the evaluated samples [10,13,14].

The antinociceptive effects of *S. hispanica* appear to involve opioid and GABAergic mechanisms and inhibition of lipoxygenases, cyclooxygenases, and other inflammatory mediators. In contrast, *C. × latifolia* compounds are associated with mechanisms such as nitric oxide (NO) production, the activation of γ-aminobutyric acid (GABA) and serotonin receptors, opioid-like effects, the inhibition of NMDA/TRPV-1 receptors, cytokine production, and oxidative stress. The antioxidant activity of both extracts likely contributes to their antinociceptive effects, particularly in the case of *C. × latifolia* [26,27,30,31,32].

The results of this study align with those reported for Chilean chia seeds, which demonstrated a total phenolic content of 0.94 ± 0.06 mg GAE/g and antioxidant activity of 0.44 ± 0.01 mg Trolox equivalent/g and 0.40 ± 0.01 mg Trolox equivalent/g using the DPPH and FRAP methods, respectively [33]. Similarly, the total phenolic content and antioxidant activity of *C. × latifolia* (14.8 ± 1.97 mg GAE/g and 37.2 ± 0.85 mg Trolox equivalent/g) were consistent with findings by Beh et al. [34], who reported a total phenolic content of 12.13 ± 1.44 mg GAE/g and DPPH antioxidant capacity of 0.313 ± 0.143 mg/mL in freeze-dried juice of *C. × latifolia*. Notably, these similarities were observed despite the plants being cultivated in Mexico and Malaysia.

This study is the first to demonstrate that the *n*-hexane extract of *S. hispanica* seeds and the freeze-dried juice of *C. × latifolia* fruit exhibit antinociceptive effects, both individually and in combination, in female rats. Recent studies investigating female animals have revealed large sex differences in areas ranging from gene-expression profiles to animal behaviors in pain sensitization. The study’s objective was not to study sexual dimorphism in synergistic effects. However, it is important to consider that the results obtained could differ in males [35,36].

## 5. Conclusions

In conclusion, oral administration of an *n*-hexane extract from *S. hispanica* seeds and the freeze-dried juice of *C. × latifolia* demonstrated dose-dependent attenuation of visceral pain in acetic acid-induced writhing tests in rats. Notably, combining these extracts at a fixed 1:1 ratio exhibited synergistic antinociceptive effects. These findings suggest that combining *S. hispanica* seed extract and *C. × latifolia* juice warrants further investigation as a potential therapeutic agent for managing visceral pain. This is particularly relevant given the widespread consumption of beverages that combine *C. × latifolia* juice with *S. hispanica* seeds.

## Figures and Tables

**Table 1:** Doses employed of *S. hispanica* and *C. × latifolia* extracts in combination.

*S. hispanica* (mg/kg, *p.o.*)	*C. × latifolia* (mg/kg, *p.o.*)	Total Dose
59.0	24.2	83.2
29.5	12.1	41.6
14.7	6.0	20.7
7.3	3.0	10.3
3.6	1.5	5.1

**Table 2:** Total soluble phenolic compounds and antioxidant activity of *S. hispanica* and *C. × latifolia* extracts.

Sample	Total Soluble Phenolic Compounds *	Antioxidant Capacity	
		Iron Reducing Power **	DPPH Method **
*n*-hexane extract of *S. hispanica*	6.6 ± 0.28 ^d^	0.39 ± 0.26 ^f^	2.13 ± 0.21 ^e^
freeze-dried juice of *C. × latifolia*	14.8 ± 1.97 ^c^	33.4 ± 2.43 ^b^	37.2 ± 0.85 ^a^

* mg equivalents to mg of gallic acid per g of extract and ** mg equivalents of mg of Trolox per g of extract. ^a–f^ Different superscript letters indicate statistically significant differences (*p ≤* 0.05). Results are expressed as mean (*n* = 6) ± standard deviation.

## Data Availability

The data presented in this study are available on request from the corresponding author due to privacy.

## Supplementary Materials

The following supporting information can be downloaded at https://www.mdpi.com/article/10.3390/nu17111884/s1: Table S1: First 20 natural products (NPs) predicted from the results of the 13C NMR-based dereplication analysis, of the n-hexane extract. Table S2. First 20 NPs predicted from the results of the 13C NMR-based dereplication analysis, of the dichloromethane extract. Table S3. First 20 NPs predicted from the results of the 13C NMR-based dereplication process, of the methanol extract. Reference [9] is cited in the Supplementary Materials.

## Abbreviations

The following abbreviations are used in this manuscript:

MeOH	Methanolic
CYP	Cytochrome
Hex	Hexane
CH_2_Cl_2_	Dichloromethane
