# Supplementary material for: An Isobolographic Analysis of the Antinociceptive Effect of Salvia hispanica L. in Combination with Citrus × latifolia in Rats
Source: Nutrients. 2025 May 30;17(11):1884. doi: 10.3390/nu17111884 (PMC12157707; doi:10.3390/nu17111884)
Supplement: Supplementary file 1 [file nutrients-17-01884-s001.zip › nutrients-3635564-supplementary.pdf]

nutrients

MDPI

Article

# An Isobolographic Analysis of the Antinociceptive Effect of *Salvia hispanica* L. in Combination with *Citrus × latifolia* in Rats

**Lilian Dolores Chel-Guerrero [1], Rolffy Ortiz-Andrade [1,*], Enrique Sauri-Duch [2], Emilio Piña-Betancourt [2], Luis Hebert-Doctor [1] and Myrna Déciga-Campos [3,*]**

1 Laboratorio de Farmacología, Facultad de Química, Universidad Autónoma de Yucatán, Mérida 97000, Mexico; ldch.guerrero7@gmail.com (L.D.C.-G.); luis.herbert@correo.uady.mx (L.H.-D.)
2 Laboratorio de Análisis Instrumental, Tecnológico Nacional de México, Instituto Tecnológico de Mérida, Mérida 97118, Mexico; enrique.sd@merida.tecnm.mx (E.S.-D.); le20081634@merida.tecnm.mx (E.P.-B.)
3 Sección de Estudios de Posgrado e Investigación, Escuela Superior de Medicina, Instituto Politécnico Nacional, Ciudad de México 07340, Mexico
* Correspondence: mdeciga@ipn.mx (M.D.-C.); rolffy@correo.uady.mx (R.O.-A.)

## SUPPLEMENTARY DATA

### Phytochemical analysis of *Salvia hispanica* L., seed extracts, based on $^{13}$C-NMR dereplication

**Table S1**. The first 20 natural products (NPs) were predicted from the $^{13}$C NMR-based dereplication analysis of the hexanic *Salvia hispanica* extract.

| Rank | Name | Score ($\delta_C$ Match) |
|---|---|---|
| 1 | Nonanoic acid | 1.0 (9/9 carbons) |
| 2 | Hexanol | 1.0 (6/6 carbons) |
| 3 | Capryc acid | 1.0 (10/10 carbons) |
| 4 | Undecanoic acid | 1.0 (11/11 carbons) |
| 5 | Heptanoic acid | 1.0 (7/7 carbons) |
| 6 | Amyl alcohol | 1.0 (5/5 carbons) |
| 7 | Hexanoic acid | 1.0 (6/6 carbons) |
| 8 | Undecane | 1.0 (11/11 carbons) |
| 9 | Octanol | 1.0 (8/8 carbons) |
| 10 | 2-hexenol | 1.0 (6/6 carbons) |
| 11 | 3-octenol | 1.0 (8/8 carbons) |
| 12 | Caprylic acid | 1.0 (8/8 carbons) |
| 13 | Myristoleic acid | 1.0 (14/14 carbons) |
| 14 | Linoleic acid | 0.1 (18/18 carbons) |
| 15 | A-linolenic acid | 0.1 (18/18 carbons) |
| 16 | Arachidonic acid | 0.95 (19/20 carbons) |
| 17 | Methyl linolenate | 0.95 (18/19 carbons) |
| 18 | Methyl octadeca-9,12,15-trienoate | 0.95 (18/19 carbons) |
| 19 | Hexyl octanoate | 0.93 (13/14 carbons) |
| 20 | Lauric acid | 0.92 (11/12 carbons) |

**Table S2**. The first 20 NPs were predicted from the $^{13}$C NMR-based dereplication analysis of the dichloromethane *Salvia hispanica* extract.

| Rank | Name | Score ($\delta_C$ Match) |
|---|---|---|
| 1 | Undecane | 1.0 (11/11 carbons) |
| 2 | Nonanoic acid | 1.0 (9/9 carbons) |
| 3 | Hexanol | 1.0 (6/6 carbons) |
| 4 | N-tridecanoic acid | 1.0 (13/13 carbons) |
| 5 | Amyl alcohol | 1.0 (5/5 carbons) |
| 6 | Capric acid | 1.0 (10/10 carbons) |
| 7 | Lauric acid | 1.0 (12/12 carbons) |
| 8 | Caprylic acid | 1.0 (8/8 carbons) |
| 9 | Undecanoic acid | 1.0 (11/11 carbons) |
| 10 | Myristoleic acid | 1.0 (14/14 carbons) |
| 11 | Hexyl octanoate | 1.0 (14/14 carbons) |
| 12 | Octanol | 1.0 (8/8 carbons) |
| 13 | Myristic acid | 1.0 (14/14 carbons) |
| 14 | Oleic acid | 0.1 (18/18 carbons) |
| 15 | Arachidonic acid | 0.1 (20/20 carbons) |
| 16 | Methyl linolenate | 0.95 (18/19 carbons) |
| 17 | Linoleic acid | 0.94 (17/18 carbons) |
| 18 | A-linolenic acid | 0.94 (17/18 carbons) |
| 19 | Pentadecanoic acid | 0.93 (14/15 carbons) |
| 20 | Methyl octadeca-9,12,15-trienoate | 0.89 (17/19 carbons) |

**Table S3**. The first 20 NPs were predicted from the 13C NMR-based dereplication process of the methanolic *Salvia hispanica* extract.

| Rank | Name | Score ($\delta_C$ Match) |
|---|---|---|
| 1 | Alpha-L-glucopyranose | 1.0 (6/6 carbons) |
| 2 | Beta-L-glucopyranose | 1.0 (6/6 carbons) |
| 3 | Mannitol | 1.0 (6/6 carbons) |
| 4 | Sucrose | 1.0 (12/12 carbons) |
| 5 | Caprylic acid | 1.0 (8/8 carbons) |
| 6 | Hexyl acetate | 0.88 (7/8 carbons) |
| 7 | (2r,3s,4s,5r,6s)-2-(hydroxymethyl)-6-{[(1s,4r,6s)-1,3,3-trimethyl-2-oxabicyclo[2.2.2]octan-6-yl]oxy}oxane-3,4,5-triol | 0.88 (14/16 carbons) |
| 8 | (2r,3s,4s,5r,6s)-2-(hydroxymethyl)-6-{[(1s,4r,6r)-1,3,3-trimethyl-2-oxabicyclo[2.2.2]octan-6-yl]oxy}oxane-3,4,5-triol | 0.88 (14/16 carbons) |
| 9 | 2-(hydroxymethyl)-6-({1,3,3-trimethyl-2-oxabicyclo[2.2.2]octan-6-yl}oxy)oxane-3,4,5-triol | 0.88 (14/16 carbons) |
| 10 | 5-methylhexanoic acid | 0.86 (6/7 carbons) |
| 11 | Heptanoic acid | 0.86 (6/7 carbons) |
| 12 | Hexyl octanoate | 0.86 (12/14 carbons) |
| 13 | Beta-D-glucopyranose | 0.83 (5/6 carbons) |
| 14 | Beta-D-glucosa | 0.83 (5/6 carbons) |
| 15 | 2-hexenol | 0.83 (5/6 carbons) |
| 16 | Hexanol | 0.83 (5/6 carbons) |
| 17 | Hexanoic acid | 0.83 (5/6 carbons) |
| 18 | Agropine | 0.82 (9/11 carbons) |
| 19 | Undecanoic acid | 0.82 (9/11 carbons) |

| | | |
|---|---|---|
| 20 | (2r,3s,4s,5r,6s)-2-(hydroxymethyl)-6-{[(1r,4s,6r)-1,3,3-trimethyl-2-oxabicyclo[2.2.2]octan-6-yl]oxy}oxane-3,4,5-triol | 0.81 (13/16 carbons) |

**Reference:**

Herbert-Doctor, L. A., Sánchez-Recillas, A., Ortiz-Andrade, R., Hernández-Núñez, E., Araujo-León, J. A., Coral-Martínez, T. I., Cob-Calan, N. N., Segura Campos, M. R., & Estrada-Soto, S. (2023). Vasorelaxant Activity of *Salvia hispanica* L.: Involvement of the Nitric Oxide Pathway in Its Pharmacological Mechanism. Molecules, 28(17), 6225. https://doi.org/10.3390/molecules28176225
